# Accuracy of the smaller superior mesenteric vein sign for the detection of acute superior mesenteric artery occlusion

**DOI:** 10.1002/ams2.313

**Published:** 2017-09-28

**Authors:** Takaaki Nakano, Toshitaka Ito, Tetsuhiro Takei, Masaaki Takemoto

**Affiliations:** ^1^ Department of Emergency Medicine Shinyurigaoka General Hospital Kawasaki Japan; ^2^ Department of Critical Care Medicine Yokohama Minato Red Cross Hospital Yokohama Japan

**Keywords:** Smaller SMV sign, Acute superior mesenteric artery occlusion

## Abstract

**Aims:**

The smaller superior mesenteric vein (SMV) sign is a well‐known computed tomography (CT) parameter for acute superior mesenteric artery (SMA) occlusion. This CT sign is potentially beneficial for the early diagnosis of acute SMA occlusion; however, few reports have documented this sign. The present study aimed to determine the accuracy of the smaller SMV sign for the detection of acute SMA occlusion.

**Methods:**

We retrospectively reviewed CT images from 20 patients with acute SMA occlusion and 1,216 controls. We measured the external diameters of the SMV and SMA, and calculated the SMV/SMA diameter ratio. A ratio ≤1 indicated a positive smaller SMV sign.

**Results:**

Of the 20 patients, 14 had the smaller SMV sign, whereas of the 1,216 controls, 88 had the smaller SMV sign. Of the 88 controls with a positive sign, 79 had apparent reasons for the decreased flow in the SMA and nine patients had no reason for the decreased flow. The sensitivity and specificity of the smaller SMV sign for acute SMA occlusion were 70% and 99.2%, respectively.

**Conclusion:**

The smaller SMV sign is an accurate and important CT parameter for the detection of acute SMA occlusion.

## Introduction

Acute superior mesenteric artery (SMA) occlusion is rare, and accounts for less than 1 of every 1,000 hospitalizations. Its mortality rate has been reported to be up to 60–80%, and a delay in diagnosis can result in life‐threatening consequences.^1^ This disease should be evaluated early, and the time to recanalization is extremely important.^1^, ^2^ There are various clinical findings in patients with this disease; therefore, diagnosis can be extremely difficult. For the diagnosis of acute SMA occlusion, imaging techniques, such as ultrasonography, computed tomographic angiography (CTA), and magnetic resonance angiography, are useful.^1^, ^3^, ^4^ However, each of these methods has limitations. Ultrasonography is highly dependent on the skill of the technologist, and it can be difficult to carry out in patients with obesity, bowel gas, and heavy calcification in the vessels. Computed tomographic angiography has a high accuracy for the diagnosis of SMA occlusion (up to 95–100%). However, there might be issues with the use of contrast agents.^5^, ^6^ Magnetic resonance angiography requires a long time for imaging.^1^ Therefore, a quick and accurate diagnostic indicator for acute SMA occlusion is required. The smaller superior mesenteric vein (SMV) sign has been often discussed in textbooks.^7^

However, there are few clinical reports on this sign.^8^ The smaller SMV sign can be identified in plain CT images, without the use of a contrast agent, therefore, it can be quickly and reliably detected.

The present study aimed to determine the accuracy of the smaller SMV sign for the detection of acute SMA occlusion.

## Methods

A total of 20 patients treated for acute embolic occlusion of the SMA between 2005 and 2014 were reviewed retrospectively. The diagnosis was confirmed based on surgical findings, CTA, or catheter angiography. The control group included 1,220 individuals who presented to the emergency department for other reasons and did not have acute SMA occlusion. The control group included 116 patients with abdominal pain. All CT studies were undertaken with a 64‐section helical scanner (pitch, 0.6; 5‐mm intervals). Computed tomography images of patients and controls were evaluated.

**Figure 1:**
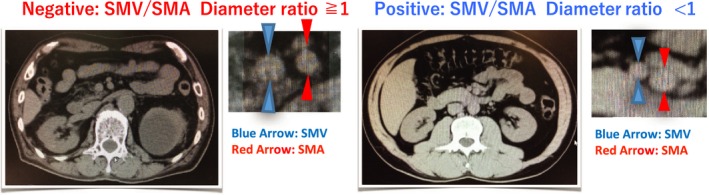
Plain computed tomography (CT) images of the smaller superior mesenteric vein (SMV) sign for the detection of acute superior mesenteric artery (SMA) occlusion. The SMA and SMV exist side‐by‐side in the upper part of the kidney. Left panels, CT images showing a negative smaller SMV sign, suggesting normal perfusion of the intestine. Right panels, CT images showing a positive smaller SMV sign, suggesting hypoperfusion of the mesenteric artery.

In plain CT images, the SMV and SMA appear side‐by‐side in the upper part of the kidney (Fig. 1). We used the smallest diameters of the vessels to determine the SMV/SMA ratio. A ratio ≥1 was considered normal, indicating the absence of a smaller SMV sign, while a ratio <1 indicated the presence of a smaller SMV sign and an acute SMA occlusion. We determined the number of patients with a positive smaller SMV sign.

Additionally, we reviewed data on medical history that could affect intestinal perfusion (SMA blood flow) and Mogi classification. The Mogi classification system is used to categorize acute SMA occlusion according to the occlusion site.^9^ Mogi class A indicates occlusion at the proximal branch of the middle colic artery, class B indicates occlusion at the ileocecal artery branch, and class C indicates occlusion at the distal portion of the SMA. We used this system because the smaller SMV sign might not be useful for detection of acute SMA occlusion at some sites.

## Results

A smaller SMV sign was identified in 14 of the 20 patients (Table 1). The mean time from onset to CT imaging was 10.5 h. Mogi class A occlusion was present in 17 patients; of these, 12 had a smaller SMV sign. Class B occlusion was present in two patients, and one had a smaller SMV sign. Class C occlusion was present in one patient, who had a smaller SMV sign (Table 2). There were no significant differences between occluded segments. In patients with a smaller SMV sign, the mean time from onset to CT imaging was 13.4 h, whereas in patients without a smaller SMV sign, the mean time from onset to CT imaging was 3.4 h; however, the difference was not significant.

**Table 1:** Characteristics of patients with superior mesenteric artery (SMA) occlusion with a positive smaller superior mesenteric vein (SMV) sign

No. of patients with acute SMA occlusion	SMV/SMA diameter ratio, mean (range)	No. of patients with a positive smaller SMV sign	Time from onset to CT, mean (range)
20	0.94 (0.53–1.47)	14 (70%)	10.5 h (1–72 h)

CT, computed tomography.

**Table 2:** Characteristics of patients with superior mesenteric artery (SMA) occlusion according to the Mogi classification

Mogi classification	SMV/SMA diameter ratio, mean (range)	No. of patients with a positive smaller SMV sign	Time from onset to CT, h; mean (range)
A (*n* = 17)	0.94 (0.54–1.39)	12	11.3
B (*n* = 2)	1.01 (0.55–1.47)	1	8
C (*n* = 1)	0.92	1	2

Class A, occlusion at the proximal branch of the middle colic artery. Class B, occlusion at the ileocecal artery branch. Class C, occlusion at the distal portion of the SMA.

CT, computed tomography; SMV, superior mesenteric vein.

**Figure 2:**
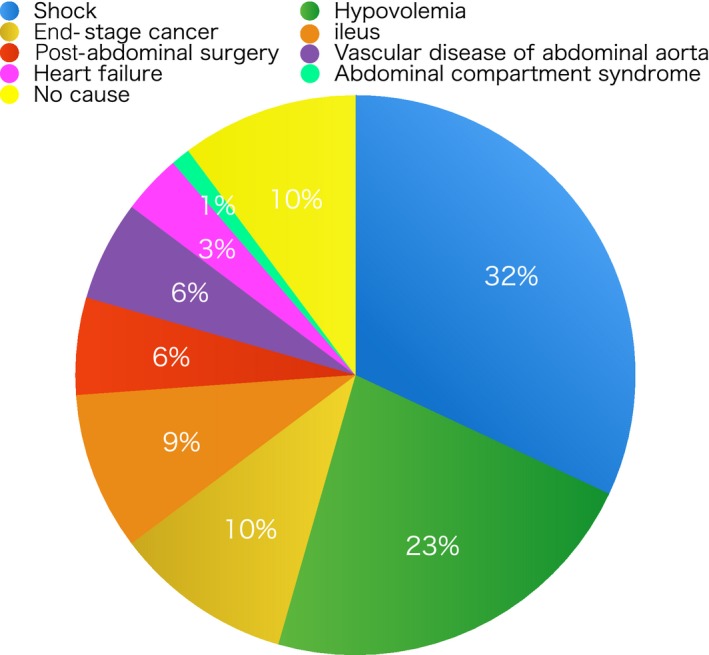
Reasons for a positive smaller superior mesenteric vein sign in 88 controls with this sign. Nine patients had no appropriate reason.

The SMV/SMA ratio could not be evaluated in four of 1,220 controls because of malnutrition and intraperitoneal bleeding. The control group included 116 patients with abdominal pain. The SMV/SMA ratio in the assessed controls ranged from 0.55 to 2.98. The average SMA/SMV ratios in the control group and in patients with abdominal pain were 1.40 and 1.48, respectively. A smaller SMV sign was present in 88 controls (Fig. 2). A smaller SMV sign was due to shock in 28 patients (32%), hypovolemia in 20 (23%), end‐stage cancer in 9 (10%), ileus in 8 (9%), post‐abdominal surgery in 5 (6%), vascular disease of the abdominal aorta in 5 (6%), heart failure in 3 (3%), and abdominal compartment syndrome in 1 (1%). In nine patients (10%; age, 32–91 years), we were unable to determine the cause of a smaller SMV sign. Among these 9, one was diagnosed with gastroenteritis, one with cartridge case ingestion into the stomach, three with injuries resulting from falls, one with abdominal pain of unknown origin, and three with other causes. Only one patient required hospitalization because of trauma.

Of the 116 CT scans of patients with abdominal pain, seven CT scans showed a smaller SMV sign: two patients had hypovolemia, two had ileus, one had shock, one had ischemic enteritis, and one had an unknown cause.

The sensitivity and specificity of a smaller SMV sign for acute SMA occlusion were 70% and 99.2%, respectively. In CT scans for abdominal pain, the specificity of a smaller SMV sign was 99.1%.

## Discussion

A smaller SMV sign is usually described in the section regarding acute upper mesenteric artery occlusion in radiology textbooks.^7^ However, few articles have discussed its definition and usefulness. Acute intestinal ischemia has been diagnosed with CTA and angiography, and endovascular treatment is often carried out prior to surgical intervention.^10^ A previous study reported that interventional radiology before surgical intervention can increase the survival rate.^11^ The amount of contrast agent used will increase owing to CTA or interventional radiology treatment. Cigarroa *et al*.^12^ reported that administration of contrast agent beyond the maximum acceptable dose (contrast agent 5 mL/kg × body weight [kg]/serum creatinine) can increase the risk of nephropathy. A high incidence of contrast nephropathy has been reported at doses >100 mL.^5^ The onset of acute renal failure in ischemic bowel disease has been reported to increase the mortality rate.^6^ Therefore, the use of a contrast agent should be limited as much as possible. A smaller SMV sign can be determined using plain CT images, and the use of a contrast agent can be avoided.

A smaller SMV sign suggests intestinal hypoperfusion and a decrease in blood flow in the SMA. These are influenced by low blood pressure, hormones, heart failure, arterial narrowing, catecholamines, and inflammation of the intestinal tract.^13^, ^14^

Of the 1,216 controls, a smaller SMV sign was noted in 88; of these, 79 had pathologies other than embolism that reduced SMA blood flow, and nine had unknown pathologies. In a previous study in 300 patients, Suzuki^8^ reported that a smaller SMV sign was identified in 4%. However, the causes for reduced SMA blood flow were not reported.

Our results showed no difference in the time from onset to CT between patients with and without a smaller SMV sign. A smaller SMV sign was present in 70%, 50%, and 100% of Mogi classes A, B, and C, respectively. Thus, the occluded vessel and the duration of ischemia do not appear to affect the presence of a smaller SMV sign. However, the number of patients in our study was small; therefore, future studies are necessary to confirm these findings.

Liu *et al*.^2^ reported that abdominal pain associated with SMA occlusion was found in 100% of patients. Therefore, the clinical finding of abdominal pain could improve the detection of acute SMA occlusion. We experienced only one patient with abdominal pain who had a false‐positive smaller SMV sign.

A smaller SMV sign is highly specific. However, it is unclear whether hormonal variation can influence intestinal perfusion in emergencies. Additional conditions that can increase intestinal blood flow might be present. Hence, the exclusion of acute SMA occlusion based on a negative smaller SMV sign might be unsafe.

After the study concluded, we experienced a patient with abdominal pain, poor peripheral circulation, and a smaller SMV sign. The patient had chronic kidney disease, and successfully underwent direct clot removal by interventional radiology without enhanced CT. Direct clot removal requires exclusion of life‐threatening conditions, such as aortic dissection.

## Conclusion

The smaller SMV sign is an important CT parameter for the detection of acute SMA occlusion, with a sensitivity of 70% and a specificity of 99.2%. This sign can be easily detected with plain CT images, allowing for early diagnosis and treatment of acute SMA occlusion.

## Disclosure

This study was approved by the clinical research committee of Yokohama City Minato Red Cross Hospital, Japan (2016‐48).

Conflicts of Interest: The authors have no conflict of interest.
